# New insights into radiolabelled siderophores for molecular imaging of bacterial infections

**DOI:** 10.1038/s44303-025-00126-7

**Published:** 2025-11-26

**Authors:** Katerina Dvorakova Bendova, Kristyna Krasulova, Barbora Neuzilova, Marian Hajduch, Milos Petrik

**Affiliations:** 1 Institute of Molecular and Translational Medicine, Faculty of Medicine and Dentistry, Palacký University, Olomouc, Czech Republic; 2 Institute of Molecular and Translational Medicine, University Hospital, Olomouc, Czech Republic; 3 Czech Advanced Technology and Research Institute, Palacký University, Olomouc, Czech Republic

**Keywords:** Biochemistry, Biological techniques, Chemistry, Microbiology

## Abstract

This perspective article aims to provide an update on current trends in the research of radiolabelled siderophores for molecular imaging of bacterial infections. It begins by explaining the importance of developing novel diagnostic tools for infections and addresses the limitations of contemporary methods, including molecular imaging. The discussion then shifts to compounds currently being studied for nuclear imaging, with a focus on radiolabelled siderophores and recent advances in their development. It also provides the latest insights into the structures of siderophores, their utilisation by bacteria and their role in bacterial metabolism, as well as potential for labelling with various radioisotopes. Additionally, it presents the use of radiolabelled siderophores, both naturally occurring and artificial siderophore derivates, for imaging of various bacterial infections.

## Introduction – need for a new diagnostic tool

In general, bacterial infections are the second leading cause of death worldwide, accounting for approximately 7.7 million deaths per year^1,2^. The five most common pathogens in these infections are: *Staphylococcus aureus, Escherichia coli, Streptococcus pneumoniae, Klebsiella pneumoniae* and *Pseudomonas aeruginosa*^2^. The situation is becoming increasingly serious with the alarming rise in antimicrobial resistance. If no precautions are taken, some researchers estimate that by 2050, the number of deaths annually caused by antimicrobial-resistant bacterial infections may exceed 10 million^3^. Data show that antimicrobial resistance is largely caused by the extensive misuse and over-prescription of antimicrobial agents. Diagnostic uncertainty, which often leads to empirical treatment, plays a major role in this situation^4^. Current diagnostic tools that provide insight into the patient’s condition and reflect their current state of health (such as C-reactive protein estimation) have contributed significantly to the reduction in antibiotic use^5^. However, not all current methods have all the requirements to diagnose bacterial infections accurately and quickly enough. For example, molecular techniques (such as polymerase chain reaction) may not be able to distinguish between simple bacterial colonisation and active infection, and traditional culture-based procedures are often time-consuming and resource-intensive^6–8^. In addition, both methods are susceptible to contamination and rely on accurate sampling, which may involve invasive procedures that pose a greater risk to critically ill patients^9,10^.

For these reasons, molecular imaging techniques are emerging as a non-invasive, time-sensitive means of rapidly diagnosing bacterial infections. With the right tracer, hybrid imaging techniques, such as positron emission tomography (PET) or single photon emission computerized tomography (SPECT) combined with either computerized tomography (CT) or magnetic resonance imaging (MRI) could not only localise the ongoing infection in the patient and provide the results on the same day as the scan, but could potentially also quantify the bacterial burden and monitor the response to the antibiotic therapy^11–13^. However, current methods do not fulfil the potential of optimal bacterial imaging tracers. Traditionally used 2-deoxy-2[^18^F]fluoro-D-glucose ([^18^F]FDG) and [^67^Ga/^68^Ga]Ga-citrate are both taken up by metabolically active tissues, including inflammatory or malignant cells, making it difficult to differentiate these pathologies from bacterial infections^14–16^. In addition, these tracers can have high non-specific background uptake in healthy tissues (e.g., the brain, myocardium or kidneys for [^18^F]FDG), which can complicate the interpretation of results at these sites^17^. A different approach to bacterial infection imaging is the radiolabelling of white blood cells (WBC). Autologous WBCs are labelled (e.g., with [^111^In]In-oxine, or the newer [^99m^Tc]Tc-hexamethylpropylenamine oxime) and then reinjected into the patient. However, this method is time-consuming and requires handling of patient blood samples, which are prone to contamination^18,19^.

Considering the limitations of available methods, the current search for novel bacteria-specific radiolabelled molecular imaging probes needs to focus on several characteristics that an optimal tracer should possess in order to provide a diagnostic method superior to conventional methods. These include, of course, high specificity for bacterial infections, allowing differentiation from other pathologies, but perhaps also some distinction between the bacterial species, such as the Gram-negative and Gram-positive bacteria, to provide an advantage in tailoring the treatment plan^20^. High sensitivity of the tracer allows detection of the disease in its early stages. High affinity for the targeted infected tissue, with rapid accumulation of the tracer at the site of interest, followed by prolonged retention, ensures low non-specific uptake in other tissues and low background signals. Such high contrast could provide a signal proportional to the infectious burden, providing a means of monitoring disease progression and response to treatment^21^. The selected radionuclide should have a half-life long enough for the procedure to be performed, while keeping radiation exposure as low as reasonably achievable. Other suitable characteristics include high metabolic stability of the tracer, non-toxicity, availability and low production costs^20,22^.

Various compounds are being tested in the search for a novel imaging tool for infections, such as radiolabelled antibodies (e.g., non-specific human immunoglobulin or antigen-specific monoclonal/polyclonal antibodies)^23,24^, antibiotics (e.g., ^99m^Tc-labelled ciprofloxacin, ^18^F-fluoropropyl-trimethoprim, ^18^F-vancomycin based radiotracers)^25–27^ in, human neutrophil peptide)^28–30^, vitamins ([^111^In]In-biotin, [^99m^Tc]Tc-B_12_-derivate)^31,32^, aptamers^33,34^, bacteriophages^35^ or diverse molecules employing bacterial metabolism (e.g., sugars, sugar alcohols, D-amino acids, N-Acetyl muramic acid derivates, para-aminobenzoic acid, nucleoside analogues, or siderophores)^36–43^.

Although the possibility of radiolabelling siderophores has been explored since the 1970s, it has received increasing attention in recent years, due to the growing popularity of molecular imaging techniques^44^. Furthermore, the proof-of-concept studies demonstrating the ability to radiolabel siderophores with gallium-68 have stimulated further siderophore research. The earlier studies revealed the possibility of imaging mould infections caused by *Aspergillus fumigatus* in this way^45–47^, and later experiments, which will be discussed further in this article, extended the application of the same principle to bacterial pathogens^48–51^.

This review aims to provide an overview of siderophores, the current understanding of their basic mechanism and their applicability to the diagnosis of bacterial infections using molecular imaging techniques and to update the state of knowledge in this field following previous reviews^52,53^.

## Siderophores: bacterial iron scavengers

Iron is an essential element that plays a fundamental role in the survival of almost all microorganisms, including bacteria. It is involved not only in their basic metabolism, growth and replication, but also in their virulence. For example, the availability of iron has a direct effect on biofilm formation and toxin production in some bacteria^54,55^. In nature, iron occurs in two oxidation states: ferrous (Fe^2+^) and ferric (Fe^3+^). While the latter is more abundant under aerobic conditions, it is insoluble in water at physiological pH, making it less bioavailable compared to the more soluble ferrous iron^55,56^. For these reasons, bacteria have developed several strategies to compete with their mammalian hosts for iron during an infectious process. These strategies can be divided into four main categories: (1) Direct iron uptake in ferrous form; (2) acquisition from host proteins such as lactoferrin, transferrin or ferritin; (3) acquisition from haem; and (4) siderophore-mediated iron uptake^57^.

**Fig. 1:**
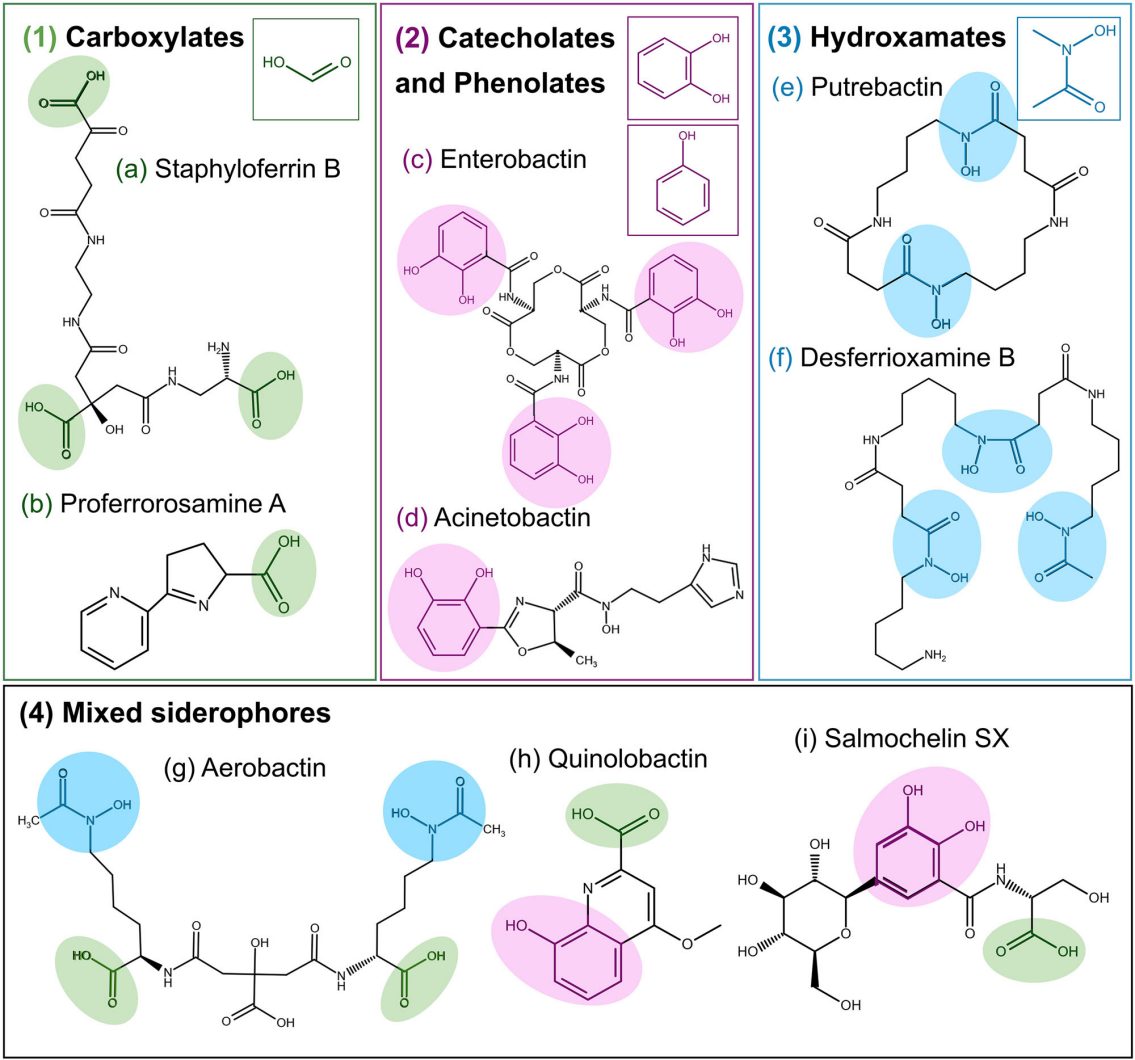
Main functional groups of siderophores and examples of bacterial siderophores for each group. Adapted from Siderite and PubChem, created in ChemSketch^59^. **(1)** Carboxylates: **a** Staphyloferrin B, **b** Proferrorosamine A; **(2)** Catecholates and Phenolates: **c** Enterobactin, **d** Acinetobactin; **(3)** Hydroxamates: **e** Putrebactin, **f** Desferrioxamine B; **(4)** Mixed siderophores: **g** Aerobactin, **h** Quinolobactin, **i** Salmochelin SX.

Iron acquisition by siderophores has been identified in both Gram-negative and Gram-positive bacteria. These low molecular weight iron chelators scavenge iron from the external environment of the bacteria or by removing it from the host proteins, as they have very high ferric-ion association constants (10^20^-10^30 ^M^-1^)^55,58^. Currently, more than 700 known structurally unique siderophores are known, and the number is constantly increasing^59^. They can be divided into 4 main categories, based on the chemical composition of their chelating moieties: (a) carboxylates; (b) catecholates and phenolates; (c) hydroxamates or (d) mixed siderophores (Fig. 1)^60^.

**Fig. 2:**
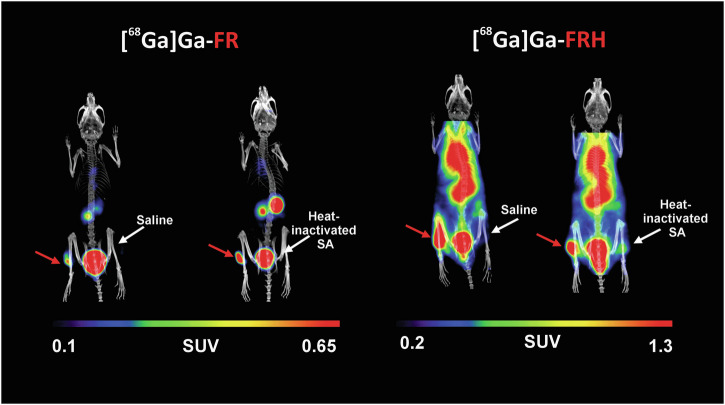
MIP PET/CT images of a mouse model of *S. aureus* myositis in the left hind leg (red arrow) using [^68^Ga]Ga-FR and [^68^Ga]Ga-FRH, while the right hind leg received a saline injection or heat-inactivated *S. aureus* (SA) culture (white arrows). These MIP images were taken 5 h after infection and 45 min after administration of radiolabelled siderophore. Previously published by Krasulova et al.^61^.

The structure of siderophores affects many aspects of the siderophore metabolism, including siderophore recognition by receptors and transporters and iron utilisation. The importance of this fact is demonstrated, e.g., by a recently published study by Krasulova et al., which discusses the effect of cis-trans isomerism of two siderophores, ferrirubin (FR) and ferrirhodin (FRH), on their radiolabelling, in vitro properties and in vivo behaviour. Although these siderophores have the same chemical formula, they differ in the 3D orientation of their acyl groups. The main findings of this study were that the nuance in their structure results in significantly higher plasma protein binding values for [^68^Ga]Ga-FRH (~50% vs. 6% after 120 min incubation), and as expected from the in vitro results, the authors observed a significantly slower pharmacokinetics of [^68^Ga]Ga-FRH and its retention in blood and most of the organs, confirmed by both ex vivo animal studies and in vivo PET/CT imaging. The mentioned study further showed that although both siderophores are produced by fungi, they exhibit significant uptake in various bacterial species in vitro. This is a practical example of siderophore piracy, which is explained later in this work. Additionally, the study evaluated the biodistribution of radiolabelled siderophores using PET/CT imaging in a murine model of *Staphylococcus aureus*-induced myositis. Myositis was induced in the left hind limb of immunosuppressed mice by injecting 50 µL of live *S. aureus* culture five hours before imaging. To assess tracer specificity, the contralateral hind limb was injected with an equivalent volume of heat-inactivated *S. aureus*. This model allows in vivo assessment of tracer uptake at both infected and control sites within the same animal, thereby minimising inter-animal variability. The use of heat-inactivated bacteria enables a direct evaluation of whether tracer accumulation reflects the presence of viable bacteria or is merely associated with inflammation induced by bacterial presence. The imaging showed clear accumulation of both ^68^Ga-siderophores in the infected tissue and no significant uptake in the heat-inactivated culture (Fig. 2)^61^.

## Unique metabolism of microbial siderophores

The metabolism of siderophores in bacteria is quite unique. The biosynthesis of siderophores depends on the availability of iron in the surrounding environment, as their production is up-regulated when iron is deficient. Synthesis occurs in three main ways in the bacterial cell, depending on the enzymes that are involved in their production. Some siderophores, such as enterobactin and salmochelins, are produced by the non-ribosomal peptide synthetase (NRPS) multienzymes; other siderophores, such as ferrioxamines and acinetoferrin, are synthesised by NRPS-independent siderophore synthetase enzymes^62–64^. However, the production of some siderophores (e.g., yersiniabactin, albomycin) involves more than one type of enzyme and can therefore be described as “hybrid” production^65^. Synthesised siderophores are then exported into the cytoplasm to the external environment, where they chelate iron and form iron-siderophore complexes.

The iron-siderophore complex is then transported back into the bacteria by specific transport systems. As the mechanism of iron-siderophore uptake has been extensively described in previous sources^66–68^, we will only provide a basic overview of the mechanism involved in iron-siderophore import, as it plays an important role in understanding the use of siderophores for bacterial imaging. In both Gram-negative and Gram-positive bacteria, the specificity of siderophore receptors varies—some are highly specific for a single siderophore, while others are more permissive and can recognise multiple structurally related siderophores. This allows some bacteria to exploit siderophores that are not produced by the bacterium itself. This phenomenon is often referred to as siderophore piracy or xenosiderophore utilisation^69^. As this strategy gives bacteria an advantage in the competition for available iron, it is used by a variety of bacterial species^70–72^. Additional details concerning xenosiderophores are thoroughly discussed in a review published by Kumar et al.^73^.

The uptake of the iron-siderophore complex differs between Gram-positive and Gram-negative bacteria due to differences in the composition of their cell walls. Gram-positive bacteria lack an outer membrane and have a cell membrane that is surrounded by a thick layer of peptidoglycan. The iron-siderophore complex is therefore bound to a siderophore binding protein (SBP), which is anchored directly to the bacterial cell membrane^74^. The complex is then transported by a permease protein in a process that is energised by ATP-binding cassettes (ABC). The ABCs provide the conformational changes that push the siderophore complex through the transmembrane channel and into the cytoplasm^75^.

**Fig. 3:**
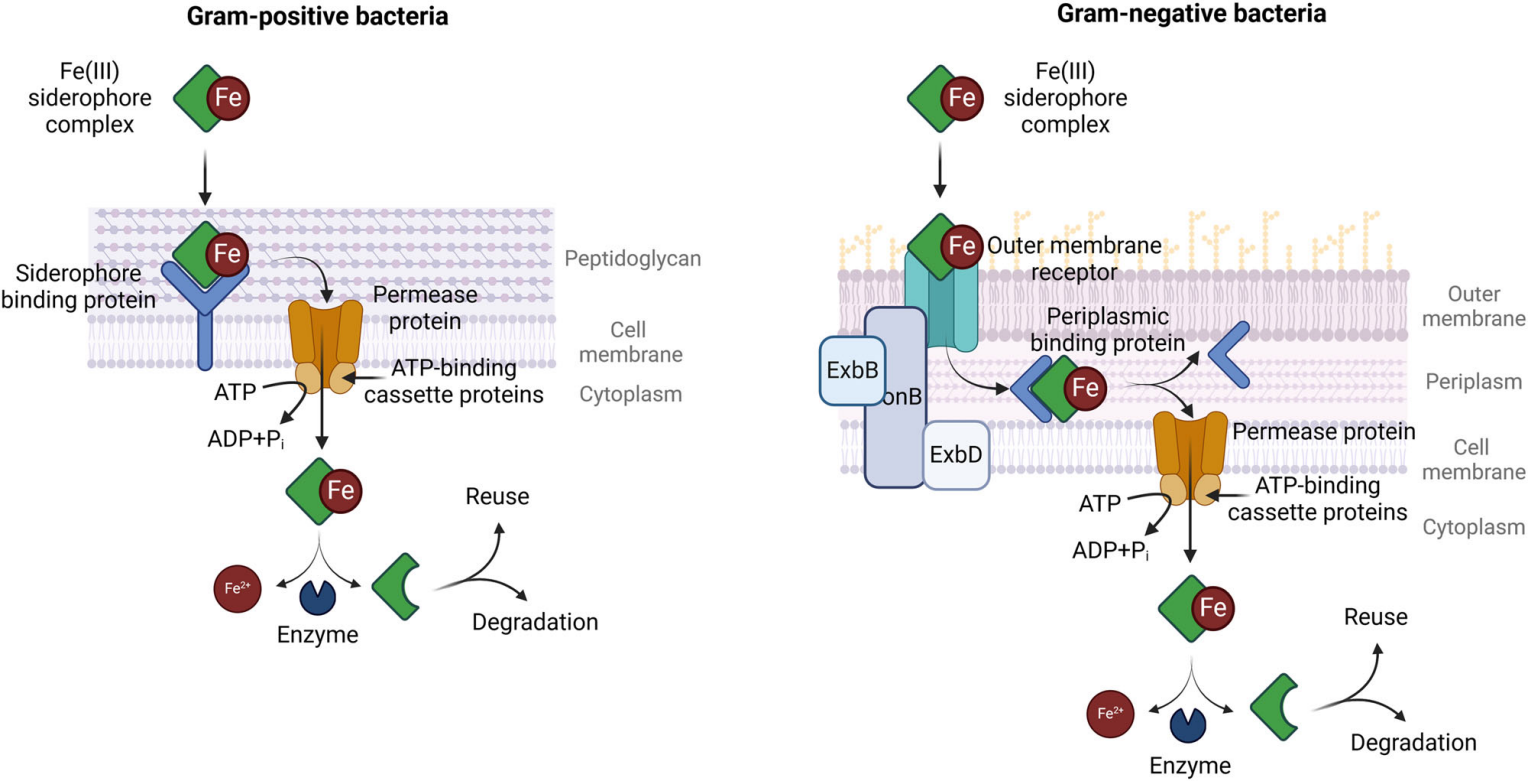
Iron-siderophore complex uptake in Gram-positive and Gram-negative bacteria. Created in https://BioRender.com. Bendová, K. (2025) (Gram-positive) https://BioRender.com/5d0c3ij and (Gram-negative) https://BioRender.com/c57m966.

In Gram-negative bacteria, the iron-siderophore complex is initially recognised by the outer membrane receptor (OMR), which interacts with the inner-membrane TonB protein. TonB is driven by other inner membrane proteins, ExbB and ExbD, which together induce a conformational change in the OMR. This alteration causes the iron-siderophore complex to be transported across the outer membrane into the periplasmic space^76^. In the periplasm, depending on the bacterial strain and siderophore type, the iron-siderophore complex is either directly cleaved or transported further into the bacteria^77^. In each case, the components are transferred across the inner membrane by the ABC-associated periplasmic binding protein (PBP), in a manner similar to Gram-positive bacteria^78,79^. The process of siderophore uptake in Gram-positive and Gram-negative bacteria is illustrated in Fig. 3.

## Siderophores as radiotracers

Due to the structural composition of siderophores and the mechanism by which they are taken up by bacteria, siderophores can be considered promising candidates for radiolabelling. The iron naturally bound to siderophores can be easily substituted by a radioisotope, providing an opportunity for delivering a selected isotope into the bacterial cell. Historically, the first experiments exploiting this principle were performed in 1980 by Emery and Hoffer, who successfully radiolabelled the ferrichrome siderophore with gallium-67. They were soon followed by Chandra et al., who labelled desferrioxamine B (DFO-B) with indium-111^44^. Both groups demonstrated that these isotopes form complexes with siderophores and that the resulting complexes retain their biological uptake into microorganisms^44,80,81^. Both gallium and indium are trivalent metal cations with similar coordination chemistry to that of iron, making them suitable substitutes in siderophores, with gallium forming slightly higher stability complexes than indium^61,82^. These isotopes have been clinically used as SPECT imaging agents since the 1970s. Both decay by electron capture with a half-life of 3.3 and 2.8 days, respectively. These longer half-lives allow for extended imaging protocols, such as monitoring infection progression or therapeutic response, but the prolonged radiation exposure to the patient must be considered^83^. Another disadvantage is that both isotopes require high-energy cyclotron production, limiting their availability compared to generator-produced isotopes^84^.

With the advances in imaging technology, the development of siderophore radiotracers expanded to PET isotopes. Gallium-68 was among the first clinically relevant PET isotopes investigated for siderophore labelling^39,45^. Its major advantage is availability from an on-site ^68^Ge/^68^Ga generator, which is fairly long-lived (T_1/2_ = 271 days) and relatively affordable. Gallium-68 has a short half-life (67 minutes), resulting in a lower radiation burden for patients. Such a short half-life does not pose a concern for siderophore imaging. These compounds usually exhibit rapid biodistribution, and significant accumulation in the targeted infected tissue has been observed as early as 5-10 minutes post-injection in mice^50,85^. Some concerns have been brought up regarding the positron energy of gallium-68^86^. Its mean positron energy (E_β+ max_ = 1.89 MeV) is higher than that of other PET isotopes (e.g., fluorine-18, E_β+ max_ = 0.63 MeV), which increases the distance between the location of the decaying parent nucleus and positron annihilation^87^. This leads to reduced spatial resolution and scan sensitivity^88^. Nevertheless, contemporary technologies involving positron range corrections and denoising techniques can decrease this effect^87^. Additionally, the PET isotope, zirconium-89, was also reported to form stable complexes with siderophores^47^. Zirconium-89 emits lower-energy positrons (E_β+max_ = 0.89 keV) than gallium-68 and has a considerably longer half-life than gallium-68 (78.4 h), making it more suitable for slow-accumulating tracers such as antibodies or nanoparticles rather than siderophores. Unlike gallium-68, zirconium is produced in a medical cyclotron, which limits its accessibility^89^. Recently, research has also been exploring the potential of alternative radioisotopes for siderophore labelling, which are discussed in respective sections.

An important aspect to consider in the research of radiolabelled siderophores and their potential for clinical translation is the availability of native siderophores. DFO-B (marketed as Desferal®) is the most notable exception, as it is widely commercially available and already used clinically, for example, for the treatment of iron overload and aluminium toxicity. A few other well-studied siderophores, such as enterobactin, staphyloferrin, or pyoverdines, can also be purchased commercially. However, many siderophores remain difficult to access, and research progress is often limited by restricted supply. Nonetheless, as interest in siderophores has increased in recent decades, numerous protocols for their acquisition have been published despite the substantial structural diversity of these compounds. In general, siderophores can be obtained either by chemical synthesis or by isolation from microbial cultures that naturally produce the compound^90–93^. Both strategies have limitations and require significant expertise, which hinders the large-scale production needed for clinical translation^94,95^. In the case of chemical synthesis, challenges include the molecular complexity of siderophores, the presence of enantiomers, and, in many cases, low yields, which increase the production cost^95,96^. Concerning isolation, the key limitations involve careful selection of siderophore-producing species, which must be evaluated for biological safety and production capacity. Furthermore, incubation conditions, particularly the formulation of the growth medium, play a decisive role, as iron availability directly regulates siderophore secretion and non-optimised conditions often lead to reduced yields. Final extraction and purification require compound-specific protocols, posing an additional obstacle for their mass production. Addressing all these challenges is crucial for enabling the full-scale translation of siderophores for clinical use, and is discussed in more detail in previously published reviews^95,96^.

In summary, the optimal choice of siderophore–isotope combination must balance several factors. The radionuclide should exhibit coordination chemistry comparable to Fe(III) to ensure efficient complexation. Radiolabelling must not compromise the siderophore’s recognition and uptake by bacterial transport systems. The resulting complex should remain stable against competing endogenous metals, even though their free concentrations in vivo are generally low^97^. Furthermore, the physical properties of the isotope—such as half-life, emission characteristics, and clinical availability—must be compatible with the pharmacokinetic profile of the chosen siderophore. In any case, the optimal siderophore-based tracer should comply with the requirements of other radiotracers, as was discussed above^20^.

## Radiolabelled natural siderophores

To explore radiolabelled siderophores' potential for bacteria-specific imaging, Petrik et al. evaluated the potential for detecting *Pseudomonas aeruginosa* infections using a gallium-68-labelled siderophore naturally produced by the bacterium, pyoverdine (PAO1). The study revealed that [^68^Ga]Ga-PAO1 behaves similarly to its ferric analogue, exhibiting high uptake in *P. aeruginosa* cultures while retaining high specificity for this pathogen. In animal models, [^68^Ga]Ga-PAO1 demonstrated excellent biodistribution, with rapid renal clearance and selective accumulation in infected muscle tissue, showing superior performance compared to clinically used tracers ([^68^Ga]Ga-citrate, [^18^F]FDG)^48^.

Siddiqui et al. investigated the possibility of radiolabelling the siderophore yersiniabactin (YbT) with a range of radiometals: ^55^Co, ^64^Cu, ^68^Ga, and ^89^Zr. The authors demonstrated that [^64^Cu]Cu-YbT has the highest complexation and showed that it is taken up by bacteria expressing the FyuA receptor, which is known to mediate the import of YbT into bacteria such as *Escherichia coli* and *Klebsiella pneumoniae*. Furthermore, the affinity of [^64^Cu]Cu-YbT for bacterial infection in vivo was demonstrated by PET imaging of a mouse model of myositis and a mouse model of pneumonia, showing accumulation of radiolabelled YbT in the tissues infected with FyuA-expressing bacteria and low signals in the negative control, *Staphylococcus aureus* or *Pseudomonas aeruginosa*, which have been reported not to express FyuA. Nevertheless, [^64^Cu]Cu-YbT displayed accumulation in non-infected organs, such as liver, heart, lung, gastrointestinal tract and kidneys, which may hinder its usability for PET imaging, and thus further improvement of the compound is required before its further application^98^.

Petrik et al. reported that the previously mentioned DFO-B could also be used to image bacterial infections using PET. The authors radiolabelled DFO-B with gallium-68 and assessed that the resulting complex had in vitro properties suitable for PET imaging. They reported high uptake in several bacterial strains, including *P. aeruginosa, S. aureus* and *S. agalactiae*. They also showed that [^68^Ga]Ga-DFO-B has optimal pharmacokinetics in healthy animals, with exclusive renal excretion. The use of [^68^Ga]Ga-DFO-B for PET imaging of infections was evaluated in two animal models of infection: acute murine myositis and acute rat pneumonia^49^. Two clinical trials with [^68^Ga]Ga-DFO-B are currently ongoing. A phase I/IIa study to evaluate the safety, biodistribution, dosimetry, and preliminary diagnostic performance of [^68^Ga]Ga-DFO-B for PET imaging in patients with bacterial infections is being performed at the Medical University of Innsbruck in Austria (EudraCT Number 2020-002868-31). Another clinical trial, also focusing on [^68^Ga]Ga-DFO-B, is currently being conducted at King’s College London (NCT number: NCT05285072) to investigate the ability of the tracer to be taken up at sites of infection in patients with vascular grafts.

**Fig. 4:**
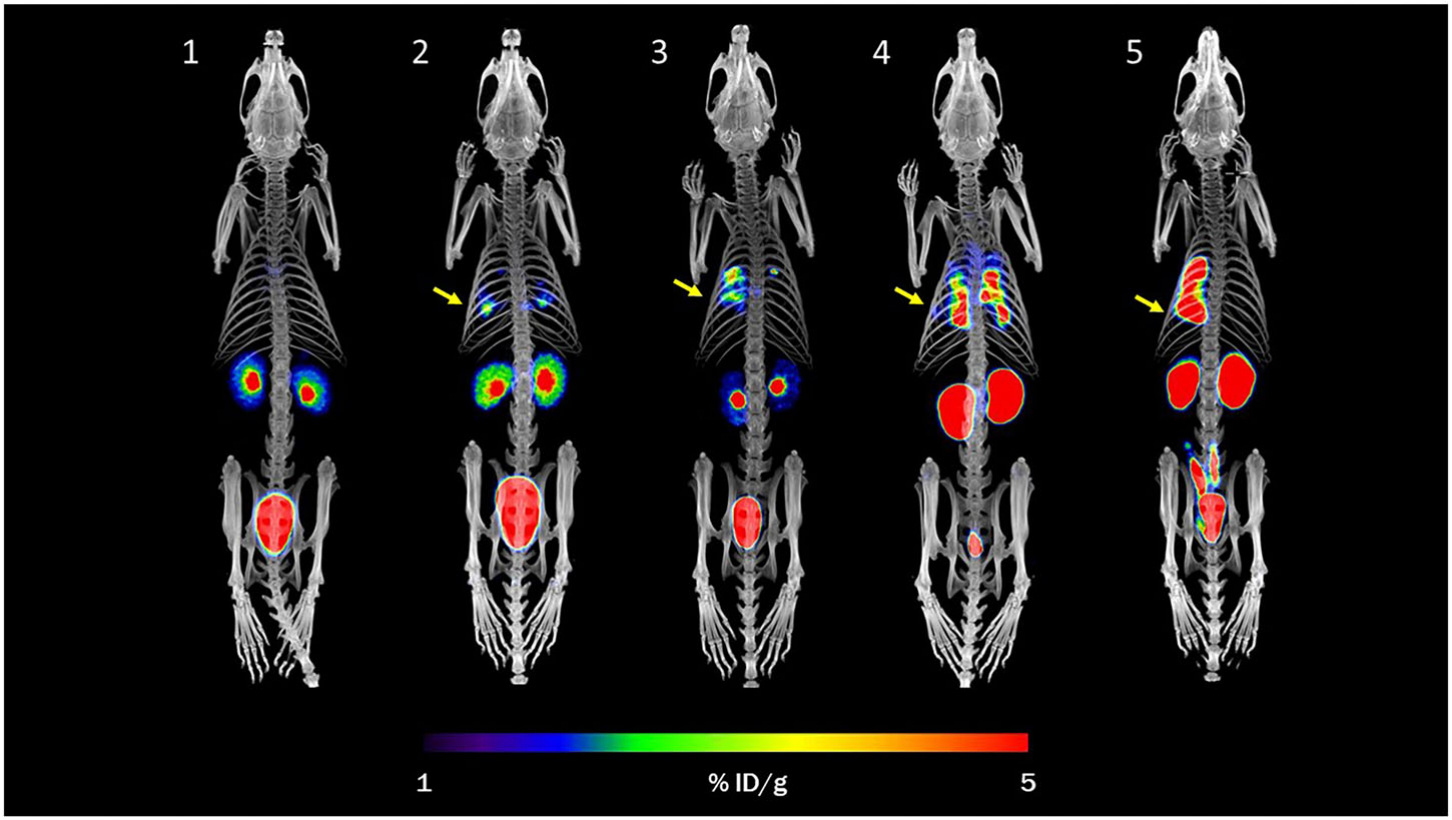
PET/CT MIP images of [^68^Ga]Ga-ORNB in a control rat (1) and in a rat model of pulmonary infection (*B. multivorans*) (2-5) 48–72 h after infection and 45 min after the injection of [^68^Ga]Ga-ORNB. Yellow arrows indicate the site of infection Previously published by Bendova et al.^50^.

A recent evaluation by Bendova et al. has assessed the potential of the radiolabelled siderophore ornibactin (ORNB) for the imaging of infections caused by the *Burkholderia cepacia* complex (BCC). The authors have successfully radiolabelled ORNB with gallium-68 with high radiochemical purity, resulting in a complex with favourable in vitro properties. They demonstrated its high in vitro specificity for BCC and optimal biodistribution in healthy mice, as it was exclusively excreted via the urinary system. In vivo PET/CT imaging of a murine model of myositis showed that [^68^Ga]Ga-ORNB can distinguish between *B. multivorans* and an *E. coli* infection or sterile inflammation. Furthermore, they demonstrated that [^68^Ga]Ga-ORNB can be used to image pneumonia in a rat model of lung infection induced by *B. multivorans* (Fig. 4)^50^.

Additionally, Dvorakova Bendova et al. investigated the potential of gallium-68-labelled siderophores for PET/CT imaging of *Acinetobacter baumannii* infections. They selected two siderophores—ferrioxamine E (FOX E) and previously mentioned FR—based on their favourable in vitro properties and significant uptake in *A. baumannii* for further evaluation. In healthy animals, [⁶⁸Ga]Ga-FR demonstrated slightly better biodistribution, with renal excretion as the primary clearance pathway. In contrast, [⁶⁸Ga]Ga-FOX E also showed minor uptake in the gastrointestinal tract. Both compounds successfully tracked *A. baumannii* infections in three distinct animal models: a murine model of myositis, a murine dorsal wound infection model, and a rat model of pneumonia. Quantitative analysis revealed that [⁶⁸Ga]Ga-FOX E exhibited slightly superior imaging results, with lower background signal and statistically significant differences between infected and control animals across all models^85^.

Akter et al. investigated the potential of using gallium-68-labelled hydroxamate siderophore schizokinen (SKN), produced by *Bacillus megaterium*, for the diagnosis of bacterial pathogens. They successfully radiolabelled SKN with gallium-68 and assessed that the resulting complex exhibited hydrophilic properties, short-term stability in human serum and uptake by several strains of bacteria: *S. aureus, S. epidermidis, E. coli* and *P. aeruginosa*. [^68^Ga]Ga-SKN also showed rapid blood clearance exclusively via the kidneys, as confirmed by both PET/CT imaging and ex vivo biodistribution studies. However, the urine analysis indicated only 22% stability of the complex. For future work, the authors plan to further evaluate the stability of [^68^Ga]Ga-SKN and explore its ability to detect infections in vivo^51^.

All natural siderophores discussed in this review are summarised in Table 1, together with key information on their microbial production, reported uptake by clinically relevant pathogens, tested radioisotopes, biodistribution profiles, and potential applications.

**Table 1:** Overview of natural siderophores investigated for radiolabelling discussed in this review.

Siderophore	Microbial producer	Xenosiderophore in clinically relevant bacteria	Modality: Tested radioisotopes	Biodistribution profiles	Potential applications	References
**Enterobactin** (ENTB)	*Enterobacteriaceae* spp. *(*such as *Escherichia coli, Klebsiella pneumoniae, Salmonella enterica)*	*Staphylococcus aureus, Yersinia enterocolitica*	**SPECT:** ^67^Ga, ^111^In **PET:** ^68^Ga	^**68**^**Ga:** Derivates demonstrate RE and moderate GITE	Microbial uptake and transport mechanisms, scaffold for radiopharmaceuticals, antimicrobial therapy	^81,104,131,132^
**Ferrioxamine B** (FOX B; DFO-B)	*Streptomyces pilosus*	*Pseudomonas aeruginosa, Staphylococcus aureus, Streptococcus agalactiae, Burkholderia multivorans*	**SPECT:** ^111^In **PET:** ^68^Ga, ^89^Zr	^**111**^**In:** Combined RE and GITE, bone marrow uptake ^**68**^**Ga:** Rapid RE, minimal retention in blood and other organs ^**89**^**Zr:** similar profile as ^68^Ga	Infection imaging, microbial uptake and transport mechanisms, scaffold for radiopharmaceuticals for targeted imaging	^44,47,49,71^
**Ferrioxamine E** (FOX E)	*Streptomyces* spp., *Erwinia spp., Pantoea* spp*., Ewingella* spp.*, Hafnia* spp., *Pseudomonas stutzeri*	*Acinetobacter baumannii, Aspergillus fumigatus, Staphylococcus aureus*	**PET:** ^68^Ga, ^89^Zr	^**68**^**Ga**: Rapid RE, moderate uptake in GIT, low blood values ^**89**^**Zr:** predominant GITE, minimal retention in blood;	Infection imaging, microbial uptake and transport mechanisms	^39,47,85,133^
**Ferrirhodin** (FRH)	*Aspergillus vesicolor, Aspergillus nidulans, Aspergillus oryzae, Botrytis cinerea, Fusarium sacchari*	*Staphylococcus aureus, Klebsiella pneumoniae, Pseudomonas aeruginosa*	**PET:** ^68^Ga	^**68**^**Ga:** Mixed RE and GITE, moderate retention in blood 90 min p. inj., uptake in liver and lung	Microbial uptake and transport mechanisms	^61^
**Ferrirubin** (FR)	*Aspergillus ochraceus*	*Acinetobacter baumannii, Staphylococcus aureus, Klebsiella pneumoniae, Pseudomonas aeruginosa, Burkholderia multivorans*	**PET:** ^68^Ga	^**68**^**Ga:** RE excretion, no uptake in non-targeted organs	Infection imaging, microbial uptake and transport mechanisms	^50,61,85^
**Ornibactin** (ORNB)	*Burkholderia cepacia* complex	Low uptake in *Pseudomonas aeruginosa, Staphylococcus aureus*	**PET:** ^68^Ga	^**68**^**Ga:** RE, minimal retention in blood and other organs	Infection imaging, microbial uptake and transport mechanisms	^50^
**Pyoverdines** (PVDs)	*Pseudomonas aeruginosa*	---	**PET:** ^68^Ga	^**68**^**Ga:** Rapid RE, minimal retention in blood and other organs	Infection imaging, microbial uptake and transport mechanisms	^48^
**Salmochelin S4** (SAL S4)	*Enterobacteriaceae spp*.	*Staphylococcus aureus, Bacteroides thetaiotaomicron*	**PET:** ^68^Ga	^**68**^**Ga:** Rapid RE, minimal retention in blood and other organs	Microbial uptake and transport mechanisms, scaffold for radiopharmaceuticals for antimicrobial therapy	^100,109,131,134^
**Schizokinen** (SKN)	*Bacillus megaterium*	*Streptococcus epidermidis, Staphylococcus aureus, Escherichia coli, Pseudomonas aeruginosa*	**PET:** ^68^Ga	^**68**^**Ga:** Predominant RE, retention in blood, GIT and ovaries 60 min p. inj.	Infection imaging, microbial uptake and transport mechanisms	^51^
**Yersiniabactin** (YbT)	*Yersinia pestis, Yersinia enterocolitica, Yersinia pseudotuberculosis, Enterobacteriaceae spp*.	---	**PET:** ^64^Cu	^**64**^**Cu:** RE and GITE, moderate uptake in heart and lung	Infection imaging, microbial uptake and transport mechanisms	^98,135^

For each compound, the microbial source, reported uptake by clinically relevant pathogens, tested radioisotopes, biodistribution characteristics, and potential applications are summarised, along with key references. *RE* renal excretion, *GIT* gastrointestinal tract, *GITE* gastrointestinal excretion.

## Radiolabelled siderophore derivates and artificial siderophores

Besides studying naturally occurring siderophores, researchers are also investigating their modifications and derivatives. One of the main aims of siderophore modification is to improve their pharmacological properties, including stability, sensitivity, and accuracy, in two main divergent directions. One approach is to extend their application to a wider range of bacterial species to develop a universal tool for bacterial imaging. The opposite approach seeks to design a pathogen-specific tool to differentiate specific bacterial species.

Ioppolo et al. attempted to develop a derivate of the previously mentioned DFO-B to slow down the clearance of [^67^Ga]-DFO, which, in their opinion, is cleared too rapidly in its natural state for imaging of infection. They succeeded in synthesising a library of [^67^Ga]-DFO derivates, some of which retained in vitro uptake in *S. aureus* cultures, while others, more lipophilic compounds, were not taken up by the bacterial culture. Subsequent biodistribution studies were performed with 4 compounds that had good uptake rates in vitro. Although they had higher uptake in infected tissues compared to non-infected tissues, they all showed high non-specific accumulation in the gall bladder, liver, and intestine, which significantly hinders their use for imaging of infection^99^.

A recent study published by Margeta et al. describes the preparation and gallium-68 labelling of a salmochelin derivate: [^68^Ga]Ga-RMA693. This derivate showed strain-dependent in vitro uptake by *E. coli*, accumulating only in the strains expressing the salmochelin receptor (IroN), which is often associated with pathogenic strains. They also showed, using in vivo PET/CT imaging and ex vivo gamma counting of mice with subcutaneous *E. coli* infection, that [^68^Ga]Ga-RMA693 accumulates in the tissue infected with *E. coli* expressing IroN. The group is continuing this work by exploring the sensitivity of the tracer and its applicability in different mouse models of infection in the currently ongoing studies^100^.

Jiang et al. synthesised deferoxamine dithiocarbamate (DFODTC) and successfully radiolabelled it with the [^99m^Tc]TcN2+ complex. The [^99m^Tc]TcN(DFODTC)2 complex showed high in vitro stability and promising in vitro uptake in *S. aureus* cultures. In an ex vivo biodistribution study in mice, the complex demonstrated moderate uptake in bacteria-infected muscle, as well as moderate uptake in turpentine-induced abscess. In addition, the complex showed increased accumulation in the healthy liver and intestine, which could potentially compromise the accuracy of imaging results. In vivo SPECT/CT imaging confirmed the ex vivo findings. While this tracer shows some potential, its uptake in the internal organs hinders its applicability to other infectious models and therefore calls for further development^101^.

**Fig. 5:**
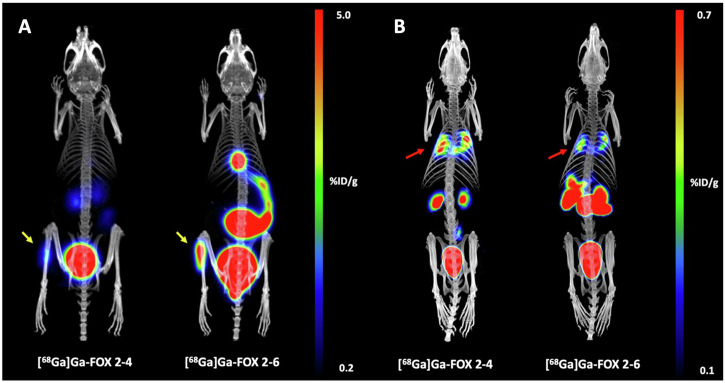
PET/CT MIP images of artificial [^68^Ga]Ga-FOXE derivatives, [^68^Ga]Ga-FOX 2-4 and [^68^Ga]Ga-FOX 2-6. **A** *S. aureus* infected mice 45 min after injection. Yellow arrows indicate the infection. **B** *A. fumigatus* infected rats 45 min after injection. The red arrow indicates the infection. Previously published by Mular et al., modified^103^.

In a different study, six derivatives of the siderophore FOX E, previously shown to be a promising radiotracer for imaging of pulmonary aspergillosis^46^, were evaluated as potential gallium carriers for therapeutic and diagnostic purposes against bacterial pathogens. All ligands were labelled with gallium-68 with sufficient radiochemical purity, and the resulting complexes showed stability comparable to naturally occurring siderophores^102^. Variable levels of uptake were observed in *S. aureus* cultures, generally correlating with growth promotion studies performed on the same bacteria. These results show that FOX E derivates have a potential for medical applications. Based on this work, further studies were carried out to compare the uptake of modified siderophores. The study showed that altering the ring size of the compounds induced species-specific uptake. These results were further confirmed in vivo, in a rat model of pneumonia caused by *A. fumigatus* and in a mouse model of acute myositis caused by *S. aureus* (Fig. 5). These results demonstrate the possibility of selective targeting of pathogens by siderophore modification and open a way for further exploration of siderophore derivatization^103^.

Peukert et al. synthesised 11 cyclen-based artificial siderophores containing catecholate units for gallium-68 labelling and investigated their uptake in bacteria. The two most promising compounds, numbered 7 and 15, showed promising results suitable for PET imaging and were then used in animal studies. Both compounds showed rapid biodistribution with significant uptake in *E. coli-*induced myositis and rapid tracer washout from lipopolysaccharide-induced sterile inflammation in the control leg. In the future, these compounds have the potential to accommodate other metal cations or undergo further modification (optimisation of properties, induction of antibacterially active moieties)^104^.

## Radiolabelled Sideromycins

**Fig. 6:**
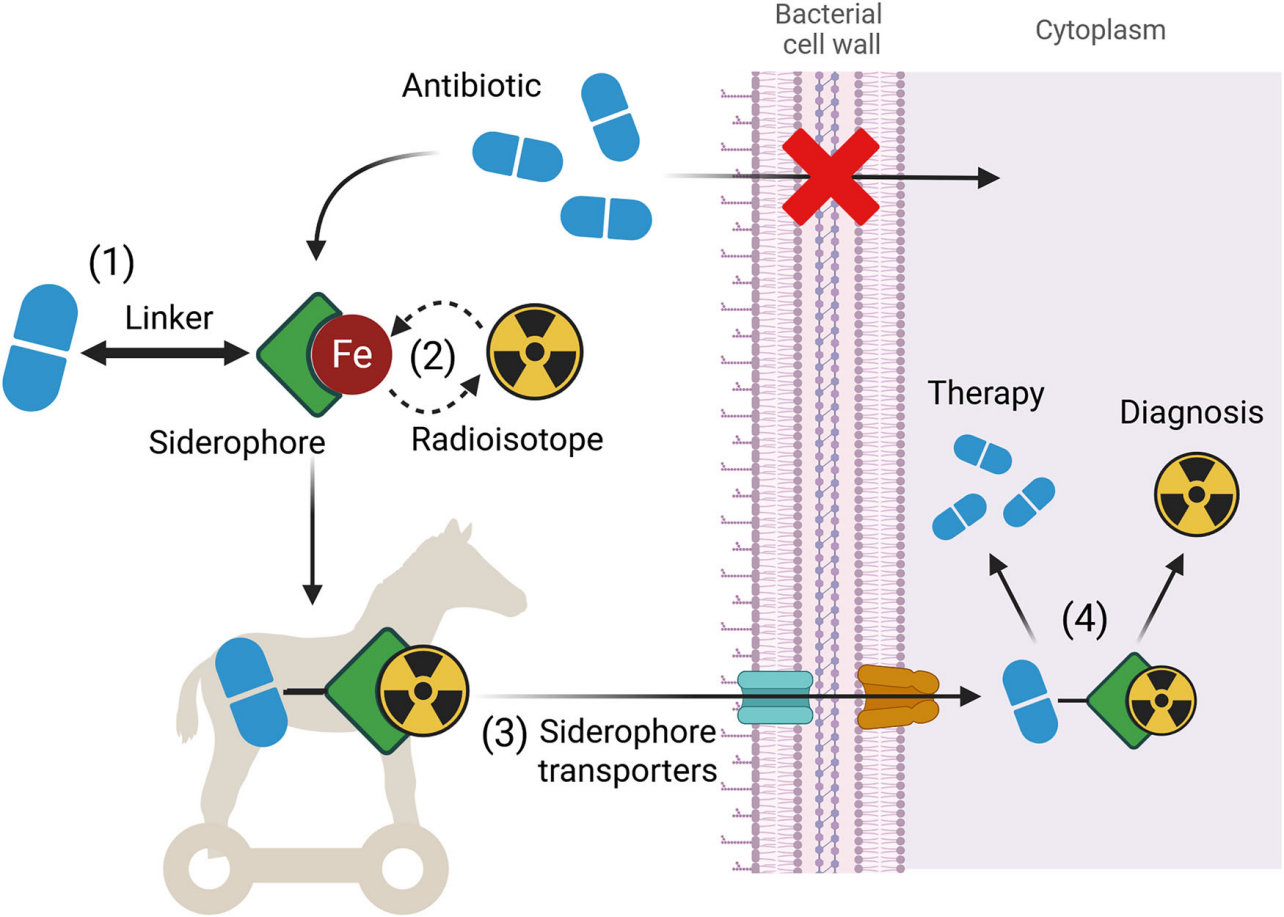
Schematic illustration of the Trojan horse strategy for targeted antimicrobial delivery via siderophores. (1) The antimicrobial agent is conjugated to the siderophores, which can be (2) radiolabelled. These conjugates are then (3) actively transported into pathogens via iron uptake pathways. Once internalised, (4) the antimicrobial compound and the radioisotope are released, enabling simultaneous detection and elimination of the microorganism (a theranostic approach). This strategy exploits specific microbial iron acquisition systems, thereby increasing drug specificity and might potentially help to overcome common resistance mechanisms. Created in https://BioRender.com. Bendová, K. (2025) https://BioRender.com/gm3sbxd.

Another primary goal of siderophore modification is to expand their potential applications beyond nuclear imaging. In such cases, siderophores are linked not only to a radioisotope but also to another functional molecule, such as antimicrobial moieties, to facilitate the import of the structure into the bacterial cell. Antimicrobial siderophores, called sideromycins, can be either naturally occurring (albomycin, salmycin) or artificial conjugates of siderophores with an antibiotic structure. This process, in which an antimicrobial molecule is transported directly into a bacterial cell thanks to its binding to a siderophore, is referred to as the “Trojan horse“ strategy, which may be a potential means of combating the global pandemic of antimicrobial resistance (Fig. 6)^105^. This fact opens up the possibility of using siderophores as theranostic tools, both for therapeutic and diagnostic purposes, which has inspired further studies. One of the earliest attempts to design such a molecule was made by Ferreira et al., who attached DOTAM (1,4,7,10-tetraazacyclododecane-1,4,7,10-tetraacetic amide) scaffold to catechol-based moieties and incorporated ampicillin for antibacterial activities^106^. Research on sideromycins was further motivated by the fact that in October 2019, the FDA approved the use of Cefiderocol, a siderophore-cephalosporin conjugate, for the treatment of urinary tract infections, followed a year later by approval for use in patients with hospital-acquired pneumonia and ventilator-associated bacterial pneumonia.

Pandey et al. aimed to design a synthetic sideromycin by attaching the ciprofloxacin molecule to desferrichrome and gallium isotopes. The conjugate they designed displayed growth-inhibitory activity in several bacterial strains with high potency in *E. coli*. In addition, the authors radiolabelled these conjugates with gallium-67 and evaluated their biodistribution and stability in naive mice through ex vivo studies^107^.

Building on their previous study, Pandey et al. sought to improve the potency of their designed compounds by synthesising a second generation of albomycin-inspired ciprofloxacin-bearing molecule. The compound, designated Galbofloxacin, exceeded the potency of the parent ciprofloxacin in vitro, and the gallium-67-labelled Galbofloxacin showed renal clearance and active uptake in *S. aureus*-infected muscle that exceeded the uptake of [^67^Ga]Ga-citrate in ex vivo studies. The therapeutic study in Balb/c mice infected with *S. aureus* resulted in survival and complete resolution of infection in mice treated with Galbofloxacin, whereas the infection was fatal after 24 h in mice treated with the same molar dose of ciprofloxacin. This research practically demonstrates the dual applicability of siderophores for theranostic purposes: on the one hand, the siderophore facilitates the transportation of antimicrobial compounds for the treatment of infection in the manner of the aforementioned Trojan horse strategy, but it also enables the diagnosis of infection thanks to its chelation of gallium-67^108^.

Sanderson et al. took a similar approach by designing a ciprofloxacin-salmochelin S4-based molecule. The main aim of the design was to selectively target bacteria that express salmochelin transporters and evade the mammalian immune response to the molecule. However, the resulting compound showed significantly lower antimicrobial activity than ciprofloxacin in *E. coli* expressing the relevant transporter. The authors radiolabelled the compound with gallium-67 in high yields and further tested its bacterial uptake. They confirmed their suspicion that the compound was not significantly taken by the bacteria tested and decided to focus their future studies on optimising the active conjugate transport^109^.

## Radiolabelled siderophores beyond nuclear imaging of bacterial infections

Although this work focuses on the use of siderophores for nuclear imaging of infection, it is important to note that their potential extends beyond this field. Since some siderophores can be conjugated with targeting molecules, their innate ability to form stable complexes with radiometals makes them versatile bifunctional chelators for additional applications. At first, the siderophore used in these cases was DFO-B, most commonly labelled with gallium-68, gallium-67, or zirconium-89 and coupled with targeting molecules, such as fibrinogen, albumin, monoclonal antibodies, nanobodies, Octreotide, or folate^110–115^. Subsequently, fusarinine C (FSC) was also used as a bifunctional chelator to couple radioisotopes (e.g., Ga-68 and Zr-89) with RGD peptide for integrin or minigastrin for cholecystokinin B receptor targeting^116–120^. All of these examples and other historical conjugates are discussed in detail in previously published articles^52,121^.

**Fig. 7:**
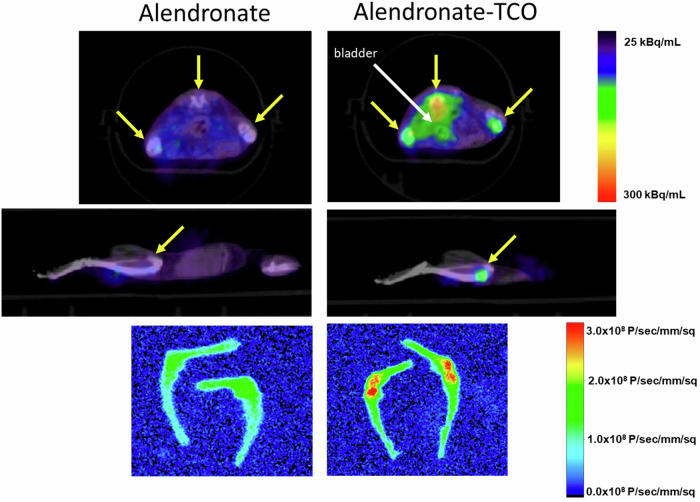
Imaging of FSC [^68^Ga]Ga-IRDdye800CW-FSC-(PEG5-Tz)2 in mice. Mice receiving bone-targeting alendronate alone (left column) or alendronate-TCO (right column), PET/CT images: transverse slices (top) and sagittal slices (middle), yellow arrows indicate uptake in joints and spine, white arrow indicates the bladder. Bottom image: Optical image of excised bones of the lower limbs. Both PET and OI indicate higher accumulation in the bone of alendronate-TCO pre-treated mice as compared to controls. Previously published by Summer et al.^122^.

In a more recent proof-of-concept study inspired by previous works, Summer et al. explored a pre-targeted imaging approach that combined optical imaging and PET. They also used the FSC scaffold for its ability to be readily radiolabeled with gallium-68. The FSC was conjugated with various fluorophores for optical imaging, as well as tetrazine moieties that selectively bind to the pretargeting agent trans-cyclooctene (TCO). In biodistribution studies conducted in mice pretreated with a bone-targeting TCO conjugate, the imaging agent accumulated in bone tissue and was clearly visualised by both PET and optical imaging, which was not observed in control animals (Fig. 7). This study clearly demonstrates the versatility of siderophores and highlights their potential application in pretargeted imaging strategies^122^.

In addition, FSC was recently used in a study that again focused on targeting cholecystokinin receptors. In this study, Gariglio et al. compared two chelating scaffolds, triazacyclononane-phosphinic acid (TRAP) and FSC, which were coupled to fluorophores and cholecystokinin binding motifs. They observed specific accumulation in tumour-bearing mice for both compounds on both PET and optical imaging, although the FSC-based molecule had more favourable biodistribution properties and higher tumour uptake^123^.

Recent research has also explored other siderophores for their chelating properties. A study by Koller et al. was inspired by siderophore-antibiotic conjugates and provides an example of an interesting alternative to the use of siderophores. The authors combined the catechol moiety from enterobactin (ENT) with a prostate-specific membrane antigen (PSMA), resulting in a new compound called ENT-DUPA. They successfully radiolabelled their new compound with titanium-45 and demonstrated that in tumour-bearing mice bearing both PSMA-positive and PSMA-negative tumours, [⁴⁵Ti]Ti-ENT-DUPA selectively accumulated in the PSMA-positive tumours and was mainly excreted via the kidneys^124^.

Interesting utilisation of siderophores was reported by Pfister et al., which further demonstrates their potential for combined imaging and theranostic applications. The team modified triacetylfusarinine C (TAFC) with several fluorescent dyes for optical imaging and labelled it with gallium-68 for PET imaging. Two of these derivates facilitated µPET/CT imaging of lung regions infected by invasive pulmonary aspergillosis, with the infected areas corresponding to lesions visualised by ex vivo optical imaging^125,126^. Additionally, the team modified the TAFC siderophore by conjugating it with various antifungal molecules. Susceptibility tests indicated potential fungal growth inhibition, and biodistribution studies in rats with pulmonary aspergillosis showed uptake of all conjugates in the infected lungs. However, in vivo studies assessing therapeutic effects have not yet been conducted^127,128^.

On the topic of *Aspergillus fumigatus*, Misslinger et al. showed that the bacterially produced DFO-B, mentioned earlier, can be used for imaging of this fungal pathogen, as they demonstrated in a rat model of pulmonary aspergillosis^129^.

## Conclusion and future perspectives

Ongoing research into radiolabelled siderophores for bacterial imaging has laid a solid foundation for future advances in targeted diagnostics. The development of these compounds holds the promise of more effective and specific imaging techniques that could significantly improve the diagnostic process for bacterial infections. In addition, the possibility of using these compounds for therapeutic purposes, such as targeted antibiotic delivery or via radionuclide therapy using suitable radionuclides^130^, represents an innovative direction in the fight against antimicrobial resistance. However, despite significant progress in radiolabelled siderophore research, their full potential has not yet been fully explored, particularly in the context of clinical applications, with a limited number of ongoing clinical trials involving these compounds.

Prospects in this field are promising, with several areas for exploration. One important direction is to further optimise the pharmacokinetic profiles of siderophore radiotracers to enhance their efficacy and reduce potential side effects. The development of new siderophore analogues that can improve binding affinity and uptake by bacterial cells is crucial. Additionally, expanding the range of pathogens that can be targeted by this imaging technique will be essential to broaden the clinical applicability of radiolabelled siderophores.

## Data Availability

No datasets were generated or analysed during the current study.
